# Effect of an Intraoral Scanner Training Program on Undergraduate Dental Students’ Self-Reported Perceptions: A Quasi-Experimental Pilot Study

**DOI:** 10.3390/dj14100641

**Published:** 2026-10-05

**Authors:** Pablo Lenin Benítez Sellán, María Paula Sánchez Rogel, Coraima Pouleth Sandoval Bravo, Antonio Lanata-Flores, Marcelo Armijos Briones, Eduardo Bresciani, Thiago Oliveira Gamba

**Affiliations:** 1 School of Dentistry, Universidad de Especialidades Espíritu Santo (UEES), Km. 2.5 vía Samborondón, Samborondón 092301, Guayas, Ecuador; mpsr@uees.edu.ec (M.P.S.R.); coraimasandoval@uees.edu.ec (C.P.S.B.); alanataf@uees.edu.ec (A.L.-F.); fernandoarmijos@uees.edu.ec (M.A.B.); 2 Centre for Rural Dentistry and Oral Health, School of Dentistry and Medical Sciences, Charles Sturt University (CSU), Orange, NSW 2800, Australia; ebresciani@csu.edu.au; 3 Department of Oral Surgery and Orthopedics, Dental School, Federal University of Rio Grande do Sul (UFRGS), Porto Alegre 91540-000, RS, Brazil; thiago.gamba@ufrgs.br

**Keywords:** intraoral scanner, dental education, digital dentistry, perception, training

## Abstract

**Background/Objectives:** Intraoral scanners (IOS) are increasingly integrated into dental curricula, yet evidence on how brief structured training affects novice students remains limited, particularly in resource-constrained settings. This pilot quasi-experimental study evaluated the effect of a structured IOS training program on the self-reported perceptions of undergraduate dental students with no prior IOS experience. **Methods:** Twenty first- and third-semester students at the School of Dentistry, Universidad de Especialidades Espíritu Santo (Ecuador), completed a 5-item Likert-type questionnaire (1–9 scale) before and after a training program comprising theoretical instruction and typodont-based practical sessions using a TRIOS 3 scanner (3Shape, Copenhagen, Denmark). Paired Student’s *t*-tests and Wilcoxon signed-rank tests were used to compare pre- and post-training scores, and effect sizes (Cohen’s dz for paired samples) were calculated for each dimension. **Results:** Significant improvements were observed in all five perception dimensions (all *p* < 0.001): familiarity (mean difference: +3.45; dz = 1.50), comfort (+3.25; dz = 1.40), confidence (+3.70; dz = 1.54), perceived scan accuracy (+3.30; dz = 1.41), and software competence (+3.55; dz = 1.30). **Conclusions:** Within the constraints of a single-group pilot design, the training program was associated with increases in students’ self-reported perceptions across all five dimensions. These preliminary findings may inform, but do not by themselves justify, decisions on the curricular integration of IOS training. Controlled studies with objective performance outcomes, validated instruments and follow-up assessment are required.

## 1. Introduction

In contemporary dentistry, the term ‘digital workflow’ describes an integrated sequence that encompasses digital image acquisition, computer-aided design (CAD), computer-aided manufacturing (CAM), and restoration delivery. Intraoral scanning (IOS) constitutes the entry point of this sequence, replacing conventional impression making as the means of capturing three-dimensional information from dental models or directly from the patient’s oral cavity [1,2]. The captured images were processed using dedicated software to accurately record the tooth morphology and surrounding tissues. The scanning accuracy is influenced by multiple factors, including moisture control, scanning distance, software algorithms, and hardware specifications [3]. Digital images are obtained with real-time visual feedback, allowing operators to identify and correct gaps or errors immediately, which is a practical advantage over conventional impression techniques that require complete remaking when defects are detected [4].

Digital and conventional impression techniques have been systematically compared in the literature [5,6,7,8]. Conventional impressions using elastomers or hydrocolloids are susceptible to dimensional variability arising from multi-step processing, manual handling and material distortion [9,10,11]. Although widely taught and practiced, these methods are technique-sensitive and depend heavily on clinician dexterity [12,13]. Intraoral scanners produce digital impressions directly, which removes several potential error sources linked to impression materials and plaster casting [14]. Research has reported comparable or superior marginal fit for restorations fabricated from digital impressions compared to conventional ones, especially for single-unit, tooth-supported restorations [5,8].

The integration of IOS into dental education has gained speed over the past decade; however, implementation remains uneven across institutions and regions [15]. Chatham et al. reported that 45% of UK dental schools had not incorporated digital impression technologies into their curricula, and that among the schools that had, only half ensured practical participation for all students [16]. More recent global survey data indicate that the adoption of digital tools varies by institutional ranking and country income level, and that intraoral scanning and CAD/CAM technologies are implemented inconsistently across institutions worldwide [17,18]. Competencies acquired during undergraduate training are critical for effective IOS use in clinical practice, which makes the design and evaluation of IOS training programmes a relevant priority for dental schools in the region [19].

Resource constraints shape IOS adoption in several concrete ways. Scanner units and their associated software licences represent a substantial capital investment relative to the operating budgets of many dental schools, which limits the number of devices available and consequently the student-to-scanner ratio achievable during teaching. Faculty development represents a second constraint, as instructors trained in conventional workflows require time and support to become confident with digital systems [20]. Where equipment is scarce, institutions must decide whether to concentrate limited access among senior students preparing for clinical practice or to distribute brief exposure more widely across the curriculum. Evidence on whether short, low-intensity training can produce meaningful change is therefore of direct practical relevance to these decisions, yet almost all available data derive from well-resourced institutions. Low-cost strategies, such as in-house three-dimensional printing combined with intraoral scanning for student self-assessment, have been proposed precisely to widen access to digital training under such constraints [21]. The present study addresses this gap by evaluating a single-session module deliverable with one scanner unit and standard preclinical infrastructure.

Prior perceptions of technology play a meaningful role in how students engage with and adopt IOS [22,23]. Students who consider digital tools clinically relevant tend to engage with training more readily and report greater confidence once they have practiced under supervision. By contrast, students accustomed to conventional impression techniques may initially be reluctant to transition to digital workflows, and some may report lower self-efficacy and a longer adjustment period [22]. This variability in initial attitudes makes it important to measure student perceptions both before and after a structured training programme, not only as an outcome measure but also to identify students who may need additional support in the future.

Currently, evidence on the relative effectiveness of different IOS training formats, including lectures, live demonstrations, typodont-based practice, and simulations, on students’ perceived competence remains limited [24]. Several investigators have reported favorable student perceptions after structured IOS curricula, yet most of these studies were conducted in well-resourced institutions with a single scanner model, which limits the generalizability of the findings [22,23,25].

Given this context, the aim of the present pilot study was to evaluate the self-reported perceptions of first- and third-semester undergraduate dental students before and after a structured IOS training program covering five dimensions: familiarity, comfort, confidence, perceived scan accuracy, and software competence. The null hypothesis was that training would not significantly change students’ ratings across these dimensions. As a pilot investigation, the study had two aims: to obtain preliminary effect-size estimates for the perception outcomes in order to inform the sample size of a future controlled trial, and to assess the feasibility of delivering the training module within the constraints of the existing preclinical timetable and equipment.

## 2. Materials and Methods

### 2.1. Study Design and Participants

This quasi-experimental pilot study enrolled 20 undergraduate dental students at the School of Dentistry, Universidad de Especialidades Espíritu Santo, Samborondón, Ecuador. First- and third-semester students were selected for two reasons. First, in the curriculum of the participating institution, formal instruction in conventional impression techniques begins in the fourth semester; students in earlier semesters therefore had no consolidated habits with alternative impression methods that might colour their appraisal of IOS. Second, digital dentistry content is not introduced until later in the programme, which made it possible to recruit a cohort with uniformly minimal baseline exposure and thus a more homogeneous starting point for evaluating the effect of the training. Given the pilot nature of the study, a formal a priori sample size calculation was not performed; a target of 20 participants was chosen to obtain preliminary effect-size estimates for future confirmatory trials, consistent with recommendations for pilot feasibility studies [26]. The study protocol was reviewed and approved by the Human Research Ethics Committee of Universidad de Especialidades Espíritu Santo (CEISH UEES) under reference code C-UEES-26-81. All procedures were performed in accordance with the Declaration of Helsinki. Prior to the commencement of the study, written informed consent was obtained from all participants. Participant data were anonymized by replacing identifiers with numerical codes. The inclusion criteria required that the participants had no prior IOS experience, ensuring comparable baseline skill levels. Students with prior IOS experience or motor disabilities that could interfere with the scanning tasks were excluded.

### 2.2. Training Program

**Figure 1:**
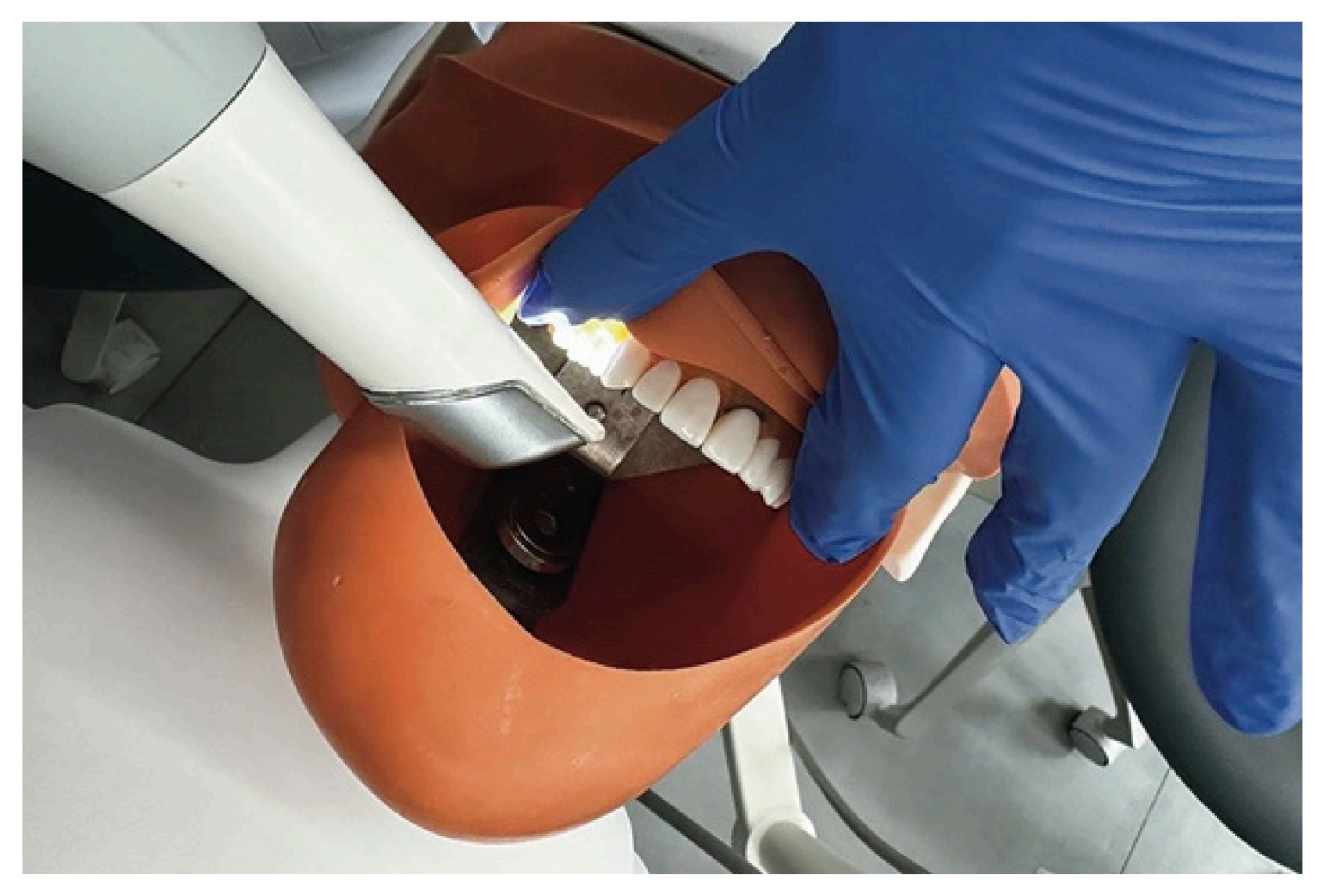
Baseline intraoral scanning on a mannequin-mounted typodont using the TRIOS 3 intraoral scanner (3Shape) during the pre-training phase.

In the pre-training phase, each student performed a baseline scan of a dental typodont (Nissin Dental Products, Kyoto, Japan) mounted on a simulation mannequin head using a TRIOS 3 intraoral scanner (3Shape, Copenhagen, Denmark) (Figure 1). The baseline scan was included as a standardised experiential reference point rather than as an outcome measure. Its purpose was to ensure that all participants had attempted the procedure before completing the pre-training questionnaire, so that their initial ratings reflected a common, concrete encounter with the device rather than an abstract expectation. Scans obtained at baseline were not scored, timed or retained for analysis, and no objective performance data were collected at any stage of the study. The training program comprised two components: (a) a theoretical component consisting of slide presentations and video demonstrations covering the operating principles, clinical indications, limitations, and scanning workflow of the TRIOS 3 system; and (b) a hands-on practical component in which participants performed supervised scans of the typodont using the intraoral scanner and associated software (3Shape, Copenhagen, Denmark), following step-by-step scanning procedures and quality-check checklists. The theoretical component lasted 10 min and was delivered in a single group session. The practical component lasted 15 min per student and was conducted with one TRIOS 3 scanner unit, giving a student-to-scanner ratio of 1:1 and a student-to-instructor ratio of 1:1, as students were scanned individually in sequential turns while the practical demonstration was shown on a per-student basis. All sessions were delivered by the same instructor, a prosthodontist with 5 years of experience in digital workflows, who supervised each participant individually during scanning. The complete programme was delivered over 2 sessions on 2 days. Infection control protocols were applied throughout the study, including the use of gloves, caps, and gowns. A scan was considered acceptable if it showed complete arch coverage without significant overlapping artifacts or unfilled voids that the acquisition software could not automatically compensate for.

### 2.3. Outcome Measures

Perceptions were measured using a purpose-designed 5-item questionnaire (Table 1) administered electronically via Google Forms immediately before and after the training program. Each item was rated on a 9-point Likert-type scale (1 = lowest and 9 = highest). The five dimensions assessed were: (P1) familiarity with IOS use, (P2) comfort with IOS technology, (P3) confidence in performing scans, (P4) perceived accuracy of scans, and (P5) self-assessed competence in using the associated software. The questionnaire was developed by the research team for the purposes of this study. The five dimensions were derived from constructs recurrently reported in previous studies of student perceptions of intraoral scanning, namely familiarity, comfort, confidence, perceived accuracy and software competence [3,22,23]. Item wording was reviewed for clarity and content relevance by three faculty members with teaching responsibilities in restorative dentistry and digital dentistry. Internal consistency of the five items was acceptable (Cronbach’s α = 0.91 pre-training; 0.80 post-training). No formal construct validation, factor analysis or test–retest reliability assessment was undertaken, and the instrument should therefore be regarded as unvalidated.

### 2.4. Statistical Analysis

All statistical analyses were performed using Jamovi (version 2.3; The Jamovi Project, Australia). Categorical variables were summarized using frequencies and percentages. Continuous variables were described using the mean and standard deviation. Normality was assessed using the Shapiro–Wilk test. Because single-item Likert-type responses are ordinal, both parametric and non-parametric approaches were applied to every comparison. Paired Student’s *t*-tests were used as the primary analysis, on the grounds that parametric procedures are robust to the ordinal nature of Likert data across a wide range of conditions, including small samples and non-normal distributions [27]. Wilcoxon signed-rank tests were conducted for all five dimensions as a sensitivity analysis. The two approaches yielded concordant conclusions for every comparison; parametric results are reported in the main text, and Wilcoxon statistics are provided in Table 2 to allow direct inspection. Effect sizes were calculated using Cohen’s d for paired samples (dz = mean difference/standard deviation of the paired differences; dz ≥ 0.8 considered large). Statistical significance was set at α = 0.05.

Generative artificial intelligence was not used to generate data, design the study, or analyze or interpret the results. Details of its use during manuscript preparation are provided in the Acknowledgments section.

## 3. Results

Feasibility outcomes were as follows. Of 35 eligible students invited, 20 consented and were enrolled (recruitment rate 57.1%). All 20 participants completed the training and both questionnaire administrations (retention 100%; item completion 100%). No session was interrupted or rescheduled, and no participant withdrew or reported discomfort during the procedure.

The sample comprised 12 women (60%) and 8 men (40%), with a mean age of 19.05 years (SD = 2.09; median age = 19 years). Ten students were enrolled in the first semester and 10 in the third semester. Semester affiliation was recorded only as an inclusion criterion and was not retained as a per-participant variable in the analysis dataset, so a formal comparison of baseline scores between the two subgroups could not be performed.

Four of the ten score distributions (five pre-training and five post-training variables) deviated significantly from normality (Table 2). For these variables, Wilcoxon signed-rank tests were conducted and yielded results consistent with those of the paired *t*-tests; therefore, parametric results are reported throughout the study.

All five perception dimensions showed statistically significant improvements from pre- to post-training (all *p* < 0.001; Table 2). The largest improvement was observed in confidence (P3: mean difference +3.70, 95% CI [2.57, 4.83]; t(19) = 6.87; dz = 1.54), followed by software competence (P5: +3.55 [2.28, 4.82]; t(19) = 5.83; dz = 1.30), familiarity (P1: +3.45 [2.37, 4.53]; t(19) = 6.69; dz = 1.50), perceived accuracy (P4: +3.30 [2.20, 4.40]; t(19) = 6.30; dz = 1.41), and comfort (P2: +3.25 [2.17, 4.33]; t(19) = 6.28; dz = 1.40). Effect sizes were large for all five dimensions (dz range: 1.30–1.54).

## 4. Discussion

Before training, the scores across all five dimensions were low. Familiarity averaged 1.90 on a 9-point scale, which was the lowest baseline value among the five dimensions and reflected the limited contact with IOS that early semester students typically have in settings where this technology is not yet part of the standard curriculum. After a single structured training session, the mean gains ranged from 3.25 to 3.70 points, with dz values between 1.30 and 1.54 for all dimensions. A comparable pattern was reported by Schott et al., who enrolled 31 dental students with no prior digital impression experience in a brief teaching module [28]. In that study, 77% of the participants rated IOS handling as satisfactory, and 58% preferred the digital technique over alginate from the operator’s perspective. Although the study design was cross-sectional rather than pre/post, the findings point in the same direction: brief, structured exposure shifts students’ appraisal of IOS toward perceived competence.

Three features of the design plausibly contribute to the magnitude of these estimates. First, baseline scores were very low, with familiarity averaging 1.90 on a 9-point scale, so that scores had considerable room to rise; effect sizes obtained from such floor conditions are systematically larger than those obtained where baseline performance is intermediate. Second, the post-training questionnaire was administered immediately after the session, when exposure to a novel technology is most salient and before any consolidation or forgetting has occurred. Third, and most importantly, the outcome was self-reported perception rather than measured performance. Perceptual self-ratings are more responsive to a single instructional episode than objective psychomotor indices, and the two should not be assumed to move together [29]. The magnitude of these effects should therefore be read as evidence of a marked attitudinal shift immediately following training, not as an estimate of skill acquisition.

The reasons why students prefer one impression technique over another are not clear. Lam et al. surveyed 97 final-year dental students across two cohorts and found that preference was driven primarily by perceived efficiency rather than by prior exposure [23]. Students who felt that scanning worked efficiently in their hands were significantly more likely to favor the digital technique (*p* = 0.000). This relationship is worth considering in light of the present study results. Of the five dimensions measured here, confidence showed the largest single gain (+3.70 points). A sense of personal confidence in performing a procedure is closely related to perceiving that procedure as efficient; therefore, the shift recorded here may have broader implications for how students approach IOS in subsequent coursework. However, whether this sense of confidence holds once students move from typodont practice to real clinical contexts requires a different study design to determine.

Studies that measure objective performance alongside student attitudes provide a useful context for interpreting perception data. In the crossover trial by Alfallaj et al. (*n* = 96 dental undergraduates), digital scanning averaged 293 s against 664 s for a conventional impression, and 76% of participants preferred the digital approach regardless of which technique they had practiced first [30]. Results from Bilir and Ayguzen, obtained in a preclinical typodont setting similar to the one used in the present study, showed a comparable time difference (272 vs. 639 s) and found that 85% of students considered the IOS method easier to handle [14]. Alfallaj et al. also observed that women completed conventional impressions faster, whereas men were quicker at digital scanning, which points to sex-related differences in psychomotor and technology familiarity patterns [30]. Because the current study measured perceptions only, without timing or objective quality data, the question of whether similar gender differences exist in the local student population cannot be addressed from these results and needs to be explored in subsequent work.

A note of caution regarding the relationship between perceptions and actual performance is warranted. Corne et al. randomly assigned 95 s-year dental students to three conditions: conventional silicone keys only, CEREC software-assisted self-assessment (version 4.4.4; Dentsply Sirona, Pennsylvania, USA), and PrepCheck pedagogic software (version 2.1; Dentsply Sirona, Pennsylvania, USA) [29]. Despite having access to digital feedback tools in two of the three conditions, no significant differences in objective preparation quality emerged, and digital feedback alone did not improve how accurately students assessed their own work. The authors concluded that constructive critique requires human reflection and that instructor guidance remains essential for converting digital feedback into skill development. This finding is directly relevant to the interpretation of the perception gains recorded in this study. The questionnaire used in the current study captured what students believed about their abilities, which was not the same as what they could objectively demonstrate. Whether the two converge after additional supervised practice remains an open question, which would require objective assessments, such as digital model superimposition against a reference, to answer properly.

For dental programs in Latin America, where institutional resources shape curriculum design in ways that differ markedly from high-income contexts, the present results carry a practical message. A one-session IOS module that combined slide-based theory with typodont scanning under supervision generated large effect sizes without requiring multi-session or high-cost infrastructure. Introducing this type of module early in training, before students have completed several clinical semesters working with conventional impression materials, may contribute to greater acceptance of digital workflows [22]. We note, however, that the present study did not measure technology acceptance, resistance to change or subsequent IOS use, and no inference about these outcomes can be drawn from perception scores obtained immediately after a single session.

### Limitations

The absence of a control group is the principal limitation. In a single-group pre/post design, the observed change is the sum of the training effect and concurrent influences that cannot be separated from it here, including maturation, a practice effect from the baseline scan, novelty of the device, and the Hawthorne effect. The effect sizes in Table 2 should therefore be read as an upper bound of what a structured session might achieve, not as an isolated training effect. A controlled design was not adopted because withholding scheduled instruction from a no-training arm was not considered defensible on educational grounds, and because enrolment was insufficient to power two randomised arms. A delayed-intervention control in a multi-centre design is the required next step.

The measurement instrument is the second limitation. The five-item questionnaire was developed for this study and underwent no formal psychometric evaluation: no factor analysis or test–retest procedure was performed, and each dimension was measured by a single item. Internal consistency was acceptable (Cronbach’s α = 0.91 pre-training; 0.80 post-training), but this cannot substitute for construct validation. Acquiescence bias and demand characteristics are both plausible given that the post-training questionnaire was completed immediately after an instructor-led session evaluating that same session, and both would inflate the observed change.

The third limitation concerns what was measured. This study assessed belief about ability, not ability: no scan execution time, retake count, software-detected errors, or trueness/precision analysis were collected. Corne et al. showed that digital feedback tools can improve student satisfaction without improving objective preparation quality or self-assessment accuracy [29], and the same dissociation cannot be excluded here. The post-training questionnaire was administered at the point of maximum salience, with no follow-up, so whether the reported gains persist is unknown. The findings are therefore best read as an immediate, context-bound reaction rather than a durable characteristic of the participants. Additional constraints include the use of a single scanner model and a sample of twenty students from one institution, which limit generalisability.

## 5. Conclusions

Within the constraints of a single-group design, this pilot study observed a marked increase in the self-reported familiarity, comfort, confidence, perceived scan accuracy and software competence of twenty undergraduate students following one structured intraoral scanning session. These are preliminary trends rather than established effects. The absence of a control group means that the contribution of the training cannot be separated from maturation, practice, novelty or observation effects, and the use of an unvalidated instrument means that the constructs underlying the scores are not psychometrically established. The sample consisted of twenty students from a single private university in Ecuador, selected from two specific semesters of one programme. Findings from a cohort of this size and composition do not support extrapolation to other institutions, curricular models, regions or resource settings, and no such extrapolation is intended. What the data do suggest, tentatively, is that a brief and inexpensive instructional module can shift how novice students appraise a technology they have never used. Whether that shift corresponds to competence, whether it endures, and whether it influences subsequent clinical behaviour are questions this design cannot answer. Addressing them requires a controlled trial with a delayed-intervention comparison group, a validated perception instrument, objective performance measures such as scan time, retake count and software-detected error rates, and follow-up assessment at a defined interval after training. Until such evidence is available, the present results should be treated as a hypothesis-generating observation from one setting rather than as a basis for curricular recommendation.

## Figures and Tables

**Table 1:** Perception questionnaire items administered before and after training.

Item	Pre-Training	Post-Training
P1. How familiar are you with the use of intraoral scanners?	1–9	1–9
P2. How comfortable are you with intraoral scanning technology?	1–9	1–9
P3. How confident are you in your ability to use an intraoral scanner effectively?	1–9	1–9
P4. How would you rate the accuracy of your scans?	1–9	1–9
P5. How competent do you feel using the software associated with the intraoral scanner?	1–9	1–9

Items were rated on a 9-point Likert-type scale (1 = lowest; 9 = highest). Both administrations used the same response format.

**Table 2:** Pre- and post-training perception scores for each dimension (*n* = 20).

Perception Dimension	Pre-Training	Post-Training	Mean Difference [95% CI]	t(19)	W	*p*	dz
Familiarity (P1)	1.90 (1.52) ^1^ 1.00 (1.00–2.25)	5.35 (2.32) 6.00 (4.00–7.00)	+3.45 [2.37, 4.53]	6.69	153.0	<0.001	1.50
Comfort (P2)	4.25 (2.05) 4.00 (3.00–5.50)	7.50 (1.32) ^1^ 7.50 (7.00–9.00)	+3.25 [2.17, 4.33]	6.28	187.5	<0.001	1.40
Confidence (P3)	3.30 (2.08) 3.00 (1.75–4.25)	7.00 (1.38) 7.00 (6.00–8.00)	+3.70 [2.57, 4.83]	6.87	202.0	<0.001	1.54
Perceived accuracy (P4)	3.80 (2.35) 3.00 (2.00–6.00)	7.10 (1.59) 7.00 (6.00–8.25)	+3.30 [2.20, 4.40]	6.30	200.5	<0.001	1.41
Software competence (P5)	3.85 (2.58) ^1^ 3.50 (1.75–6.00)	7.40 (1.35) ^1^ 8.00 (7.00–8.00)	+3.55 [2.28, 4.82]	5.83	201.0	<0.001	1.30

Scale: 1 (lowest) to 9 (highest). Data are presented as mean (SD), with median (IQR) below. Mean difference = post-training minus pre-training score, with the 95% confidence interval in brackets. t = paired Student’s *t*-test statistic (df = 19); W = Wilcoxon signed-rank statistic (sensitivity analysis); dz = Cohen’s d for paired samples (mean difference/standard deviation of the paired differences). ^1^ Shapiro–Wilk *p* < 0.05 (non-normal distribution). SD = standard deviation; IQR = interquartile range; CI = confidence interval.

## Data Availability

The raw data supporting the conclusions of this article will be made available by the authors on request.

## Abbreviations

The following abbreviations are used in this manuscript:

IOS	Intraoral scanner
SD	Standard deviation
